# RNA Interference for Mosquito and Mosquito-Borne Disease Control

**DOI:** 10.3390/insects8010004

**Published:** 2017-01-05

**Authors:** Paul M. Airs, Lyric C. Bartholomay

**Affiliations:** Department of Pathobiological Sciences, University of Wisconsin-Madison, Madison, WI 53706, USA; airs@wisc.edu

**Keywords:** RNAi, RNA interference, mosquito control, vector control, *Aedes aegypti*, *Anopheles gambiae*

## Abstract

RNA interference (RNAi) is a powerful tool to silence endogenous mosquito and mosquito-borne pathogen genes in vivo. As the number of studies utilizing RNAi in basic research grows, so too does the arsenal of physiological targets that can be developed into products that interrupt mosquito life cycles and behaviors and, thereby, relieve the burden of mosquitoes on human health and well-being. As this technology becomes more viable for use in beneficial and pest insect management in agricultural settings, it is exciting to consider its role in public health entomology. Existing and burgeoning strategies for insecticide delivery could be adapted to function as RNAi trigger delivery systems and thereby expedite transformation of RNAi from the lab to the field for mosquito control. Taken together, development of RNAi-based vector and pathogen management techniques & strategies are within reach. That said, tools for successful RNAi design, studies exploring RNAi in the context of vector control, and studies demonstrating field efficacy of RNAi trigger delivery have yet to be honed and/or developed for mosquito control.

## 1. Introduction

Insecticide resistance to DDT was originally documented in *Aedes*, *Culex*, and *Anopheles* target species less than a decade after the introduction of DDT for public health campaigns in Europe and the USA [1,2,3,4]. This trend has continued the world over for every class of chemical insecticides (pyrethroids, organochlorines, organophosphates, carbamates), with documented resistance in hundreds of mosquito species across sixty countries [5,6,7]. To combat the proliferation of pesticide resistant mosquito vectors and continue to effect control of mosquito vector-borne diseases, Integrated Vector Management and Integrated Vector Borne Disease Management programs will require alternatives to chemical pesticides [7,8].

RNA interference (RNAi) may lend itself to the cause through the suppression of gene products involved in key physiologies that impact mosquito survival, fecundity, behavior, or vector status. The RNAi pathway functions as a powerful subcellular anti-viral mechanism that post-transcriptionally suppresses mRNA transcripts based on sequence complementarity to double-stranded RNAs (dsRNAs) [9]. The pathway can be manipulated to suppress a given target gene by virtue of introducing a complementary dsRNA to mosquito cells. With hundreds of documented effective RNAi triggers targeting mosquito and pathogen genes, there is an expansive arsenal of anti-vector and anti-pathogen targets that could be harnessed for mosquito and mosquito-borne disease control strategies. RNAi experiments in a number of *Aedes*, *Anopheles*, *Culex*, and *Armigeres* disease vector species have resulted in disruption of processes including: morphogenesis, olfaction for host seeking and oviposition, blood feeding, fertility, fecundity, and survival [10,11,12,13,14,15,16,17,18,19].

**Figure 1:**
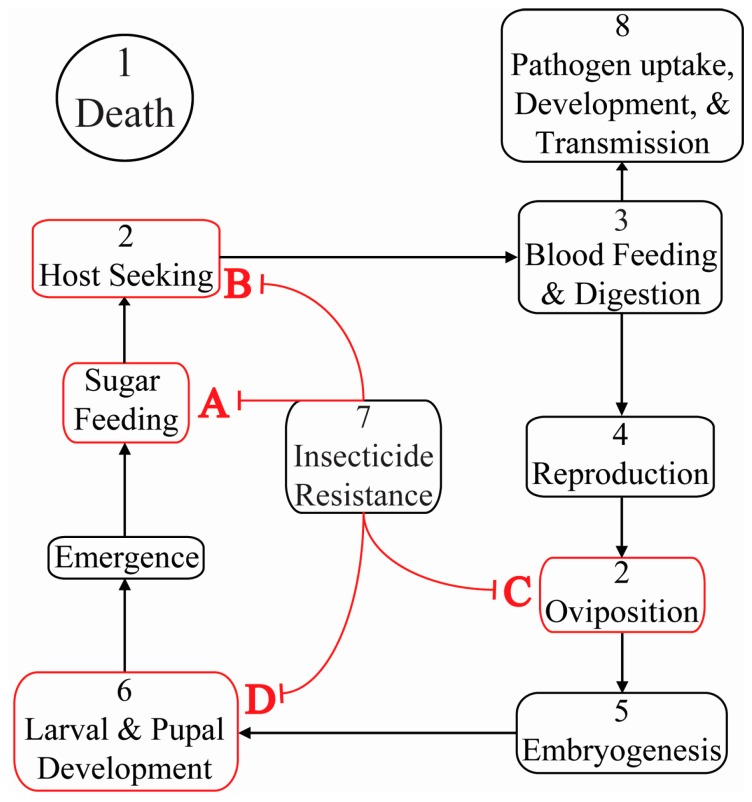
Mosquito life cycle events in the context of RNAi interventions (see also Table 1). Key life stage events illustrated in the context of targeted RNAi in mosquito disease vectors (1–8). Existing frameworks for mosquito control are shown in the context of mosquito life events to highlight points where RNAi triggers can be delivered. Control measures for particular life stages include: (**A**) Attractive Toxic Sugar Bait (ATSB); (**B**) residual spray & Long Lasting Insecticidal Bed-nets (LLIN); (**C**) Attractive Baited Oviposition Trap (ABOT); and (**D**) larvicides and pupacides. All of these control measures are at risk of resistance development (red arrows). Numerals in the figure correspond to Section 2 of the text and Table 1.

In order to achieve these same detrimental phenotypes in wild populations, RNAi triggers must be delivered to the target species and life stage with consideration for environmental and abiotic factors including: UV, ribonucleases, microbes, dissipation and dilution in aqueous environs and on solid substrates [20,21,22,23]. RNAi triggers must also be delivered to target mosquito species using field feasible applications. Infrastructure and techniques for a variety of interventions already exist to deliver chemical and biological pesticides to vector mosquitoes. Existing intervention frameworks include topical and contact applications for adults (e.g., aerial and residual spraying and long-lasting insecticidal nets (LLINs)) and *per os* or contact applications for aquatic stages (see Figure 1) [24,25,26]. RNAi knockdown in larvae by *per os* exposure is efficacious using scalable bacterial and yeast expression systems, demonstrating potential for RNAi in larval control applications [13,27]. Novel interventions have also been explored to provide oral applications to adults in the form of Attractive Toxic Sugar Baits (ATSB) [28,29,30,31,32,33,34,35,36,37]. Formulations for ATSBs include simple sucrose solutions and complex mixtures of fruit sugars with minimal effects on non-target organisms [30,36,38]. Formulations can be delivered either via spraying on plant sources or in bait stations. Surprisingly high ATSB efficacy has been found in spray formulations on flowering and non-flowering plants in arid and wet climates [30,31,35,36,39]. Additionally, strategically placed ATSB stations near breeding sites (dubbed Attractive Baited Oviposition Trap, ABOT) or indoors can attract and kill vector species in proximity to people [32,33,40,41]. Although ATSB have not been studied in conjunction with RNAi, successful gene silencing by oral exposure routes has been documented using sucrose meals and artificial blood meals demonstrating the potential in combining these control approaches [11,42]. By the same logic, mosquitocidal RNAi triggers could be applied to target essential genes for embryogenesis in Attractive Baited Oviposition Traps (ABOTs) [43,44,45]. In both baited strategies and more traditional insecticidal delivery approaches (ultra-low volume or residual sprays, or LLINs), RNAi triggers may be more efficacious in combination with biotic (e.g., a virus, yeast or bacterial expression system) or abiotic (e.g., nanoparticle) systems that mediate both protection and uptake of RNAi triggers [13,27,46,47,48,49,50,51].

Despite these knowledge and application gaps in field-relevant delivery systems, RNAi triggers offer vastly improved species-specificity with diminished environmental toxicity compared with chemical pesticides. The majority of chemical insecticides have neurotoxic activity with potential safety implications for a diverse array of organisms including beneficial arthropods, fish, and mammals [60,61,62,63,64]. By comparison, RNAi has the capacity to be as specific as the gene or sequence targeted. As such, RNAi triggers can even target specific splice variants within the target organism as demonstrated in silencing the *doublesex* sex dependency gene [13]. While there are numerous conserved sequences shared between distantly-related species, even ancient genes contain enough variation to diminish cross-species knockdown if an RNAi trigger can be designed to avoid regions with >21 contiguous matching nucleotides. For instance, in *Drosophila* species the *γ-tubulin 23C* gene shares up to 96% sequence identity but no cross-species mortality occurs following exposure to RNAi triggers designed for a specific species of *Drosophila* [65]. Likewise no cross-species mortality was found for RNAi triggers for *vacuolar ATPase* in *Drosophila melanogaster, Manduca sexta, Tribolium castaneum,* and *Acyrthosiphon pisum* [65]. Although further investigation and testing of cross-species effects should be performed, conceptually, control RNAi triggers can be designed around sequences unique to the target, with specificity far greater than chemical insecticides.

## 2. RNAi Triggers with Potential Mosquito Control Applications 

### 2.1. RNAi to Induce a Lethal Phenotype

There is great interest in developing RNAi to act as direct alternative to chemical insecticides by virtue of suppressing essential genes leading to RNAi-induced mortality. The most direct pathway to inducing mortality is via manipulation of programmed cell death. Apoptosis in insects is regulated by the Inhibitor of Apoptosis Protein 1 (IAP1) [66]. IAP1 primarily functions in inhibiting caspases and thereby preventing a cascade of subcellular events that result in degradation of hundreds of cellular components and subsequent cell death [66,67,68]. In *Ae. aegypti*, knockdown of IAP1 by RNAi results in activation of apoptosis and rapid mortality both in vitro and in vivo, making the gene a key target for insecticidal RNAi [10,52,53,54,55]. Mortality induced by IAP1 silencing can be reversed if initiator caspases such as *Ae. aegypti dronc*, which IAP1 inhibits, also are suppressed [10]. In a screen of 109 genes in *An. gambiae*, IAP1 was noted as one of two genes for which suppression significantly reduced cell growth and viability in vitro [69]. The other gene identified in this study was a ubiquitin-like/ribosomal fusion gene (*AGAP008001*). Interestingly, a similar ubiquitin gene was found to be strongly linked to apoptosis and cell survival in a genome wide screen of *D. melanogaster* [70]. In total, the *D. melanogaster* screen revealed 438 dsRNAs that induced apoptosis in vitro. This screen both validated known knockout lethal phenotype genes and identified novel essential genes including the ubiquitin-like *Ubi-p63E* and *CG11700* genes as well as DNA binding proteins *bss* and *CG15455* [70]. Beyond IAP1, apoptosis-associated lethal phenotype genes are relatively unexplored in mosquitoes. Considering that at least half of all *D. melanogaster* genes have orthologs in *An. gambiae*, there likely is a cache of highly potent, mortality-inducing target genes in mosquito species [71].

Beyond apoptosis, other essential ubiquitously and constitutively expressed genes are targeted for insecticidal activity such as tubulins and Vacuolar ATPase proton pumps (V-ATPases). Tubulin suppression by RNAi induces high levels of mortality in a diverse array of insects including *Ae. aegypti*, *Drosophila* species, *Blattella germanica*, *Diabrotica virgifera virgifera*, and *Rhodnius prolixus* [12,65,72,73,74]. In *Ae. aegypti*, mortality is induced by soaking larvae with a dsRNA targeting *β-tubulin* without transfection [12]. RNAi-based suppression of another cytoskeletal element, *actin*, in *Ae. aegypti* larvae has also led to increased Cry11Aa toxin sensitivity [75].

V-ATPase mutations have long been associated with lethal phenotypes in *Drosophila* and as such are considered vital to maintaining membrane proton translocation [76,77]. RNAi knockdown of various V-ATPases in *Drosophila*, *M. sexta*, *T. castaneum*, *A. pisum*, *Bactericerca cockerelli*, *Helicoverpa armigera,* and *D. virgifera virgifera* all result in death of the insect [65,73,78,79]. Thus far, there are several reports of V-ATPase silencing in mosquitoes with mixed results. The impact of V-ATPase subunit silencing appears to differ based on subunit, exposure route, phenotypic assay, and observed knockdown. Kang et al. (2014) explored the impact of V-ATPases on Dengue virus in *Ae. aegypti* midguts, and noted a dramatic reduction in virus titer following suppression of V-ATPase subunits by RNAi [80]. In addition to reduced Dengue virus titer, suppression of *V-ATPase subunit V0B* by injection resulted in 80.5% knockdown (72 h post exposure) leading to reduced longevity (measured over 50 days), fecundity (98% reduction), and fertility (19% reduction). Therefore the reduction in virus may be due to an overall impact on mosquito health following V-ATPase silencing. Conversely, Coy et al. (2012) showed that dsRNA delivered orally suppresses *V-ATPase subunit C* by 60% (168 h post exposure) without any noticeable death [11]. Death may have not been observed by Coy et al. (2012) because animals were monitored only for 48 h following exposure (as compared to 50 days observed by Kang et al. (2014)) [11,80]. RNAi suppression of V-ATPases in mosquito larvae also leads to adverse health outcomes. For instance, RNAi-based *V-ATPase subunit β* suppression increased Cry11Aa toxin hypersensitivity in *Ae. aegypti* larvae [75]. Additionally, using a short hairpin RNA (shRNA) Densovirus expression system to silence *ATPase subunit A* significantly reduced the lifespan of *Ae. albopictus* larvae [81]. Variation in the target subunit could explain the variation of these results because not all V-ATPase genes are considered essential. A genome- wide screen in *Drosophila* identified 33 distinct V-ATPase genes, with many subunits having multiple copies or splice variants [77]. Of these genes, only those associated with plasma membranes are known to have lethal knockout phenotypes. As such, V-ATPase as an RNAi target can produce rapid mortality, but may require RNAi triggers designed to multiple, non-redundant, or essential subunit sequences.

Finally, death can also be a by-product of suppression of genes with roles in processes beyond cell death and cytoskeletal structure. For instance, suppression of *An. gambiae* Serpin2 (SRPN2), which functions in processing prophenoloxidase, proves lethal to older mosquitoes and could be useful as a late life insecticide [78]. An RNAi trigger for prophenoloxidase III in *Ar. subalbatus* induced significant mortality and gross deformities in eclosing adults. COatamer Proteins (COPs) are required for vesicle formation and nutrient trafficking in the midgut. RNAi suppression of COPs induces death in *H. armigera* and *Ae. aegypti* [79,82,83,84]. And RNAi suppression of chitin synthase I in *Ae. aegypti* larvae is lethal during development [19,49,85]. Ultimately, it is very likely that suppression of genes with any number of functions will produce a lethal outcome, but the immediacy and potency of insecticidal RNAi triggers will differ dramatically depending on RNAi design, delivery strategy and timing, and the particular target physiology [86].

### 2.2. Olfaction

Olfaction is essential for hematophagy and associated fundamental mosquito behaviors including host-seeking and oviposition. Disruption of olfactory processes is a particularly useful approach to repelling a host-seeking mosquito—witness DEET, the most widely used active ingredient in repellent formulations on the market [87,88]. There are hundreds of chemosensory odorant (OR) and gustatory receptors (GR), ionotropic glutamate (IR) receptors, and odorant binding proteins (OBP) characterized in vector species including *An. gambiae*, *Ae. aegypti*, and *Cx. pipiens* [89,90,91,92,93]. RNAi knockdown of key sensory receptors impacts numerous essential behaviors of interest for mosquito control. For instance, silencing GR1 & 3 limits CO_2_ detection required for host-seeking [15]. Beyond host-seeking, rapid probing and blood meal engorgement significantly decrease as a result of RNAi-based suppression of OR8 and OR49 in the stylet neurons of *Ae. aegypti* [94]. In *Ae. albopictus*, RNAi knockdown of OR7 results in reduced blood feeding success and loss of human host preference in a human or mouse choice experiment [95]. Olfaction also is essential for responding to ovipositional cues. In *Cx. pipiens*, RNAi suppression of OR37 & OR99 results in reduced egg raft production and ability to sense the stimulant 4-ethylphenol [96].

Suppressing olfaction genes may offer non-chemical approaches to altering blood-feeding behavior and oviposition with clear public health benefits. However achieving this goal requires better understanding of the sequence diversity and evolution rates of olfaction genes in wild mosquito populations. As mentioned above, mutations in the *orco* gene can alter the repellency effect of DEET in *Ae. aegypti* demonstrating the potential loss of efficacy that can occur through odorant receptor mutations [87]. Another potential hurdle for an RNAi-based intervention targeting olfaction may be the increased dosage required to suppress genes in the head and antennae. In a study by Das et al. (2008) “7–8 times more dsRNA” was required to achieve gene suppression in the antennae compared to the carcass [97].

### 2.3. Blood Feeding & Digestion

Blood feeding stimulates differential expression in 50% (2388 upregulated transcripts) of all genes in *An. gambiae* [98,99]. Disruption of any of these genes immediately post-feeding could disrupt co-ordination of digestion, diuresis, can hamper oogenesis, or cause death. Upstream of digestion, suppression of genes required for probing and feeding success can block successful blood feeding. In *Ae. aegypti*, suppression of the *aegyptin* gene results in drastically reduced blood meal uptake and egg output [17]. Additionally RNAi silencing of circadian rhythm (*timeless*, *chryptochrome 1*, *takeout 1–3*) odorant receptor genes have been linked to reduced blood feeding success in *An. gambiae* and *Ae. aegypti* respectively [94,97]. After blood feeding, suppression of genes that encode digestive proteins including *gbf1*, *gap1*/*gap2*, *late trypsin*, *AaSPVI*, and *AaSPVII* in *Ae. aegypti* result in impaired oogenesis but not mortality [83,100]. Other digestive genes including *COPI* and *ARF1/ARF4* induce rapid mortality following a blood meal indicating their importance in digestion [82,83].

In addition to digestion, impairing diuresis via suppression of *arginase* and *urate oxidase* delays vitellogenesis and thereby stalls oogenesis following a blood meal in *Ae. aegypti* [101]. Overend et al. (2015) state the importance of diuresis with “genes that show conservation…in the Malpighian tubules of *Anopheles* and *Drosophila* are likely to be essential for survival” [102]. Indeed, RNAi induced suppression of diuresis via *aquaporin* suppression increases fluid retention resulting in mosquito tolerance to desiccation improving survival under stress [103,104].

### 2.4. Reproduction

Beyond inducing rapid mortality, the next logical course to reduce vector populations is to interfere with sexual development and reproduction. Preventing oogenesis, diminishing the number of female adults through sexual dependency genes, and producing sterile males are viable approaches to population control. Numerous genes limit egg batch size and nutrient uptake through disruption of nutrient regulation and developmental processes in the oocyte [18,27,56,83,100,105,106,107,108,109]. For instance, targeting genes such as *kir* in *An. gambiae,* Catalase 2 in *Ae. aegypti,* and *Cx. pipiens* (Insulin like peptides) adult females can have no impact on survival but significantly reduce egg output or egg length [107,110,111]. In these studies, knockdown of target genes persisted for 3–11 days post exposure, meaning oogenesis can be interrupted even if a mosquito takes a blood-meal many days after exposure.

Fecundity and sex bias also have been heavily investigated as a means of vector control. Sterile males can be generated via suppression of testis specific genes such as *zero population growth* or *transglutaminase* genes, which function in sperm development and storage respectively [14,112]. In a study by Whyard et al. (2015) [13], feeding larvae *E. coli* expressing dsRNAs targeting multiple male testes genes resulted in sterility in 92% of emerging male adults. This study also suppressed the female splice variant of the mosquito sex dependency gene *doublesex* in larvae, with the result that 97% of emerging adults were male. Suppression of other sex dependency genes such as *transformer 2* using an inducible RNAi-plasmid transformation was also found to result in 70% male progeny following mating of transformed adult males and females [113].

### 2.5. Embryogenesis

Embryogenesis is a transcriptionally intensive process. In *Ae. aegypti*, 8400 genes have altered transcript levels, and in *An. gambiae* the embryo contains more differently transcribed sequences (624–1009) than any other life-stage [114,115]. The embryo is an attractive life stage for delivery of RNAi triggers because cell uptake in progenitor cells conceivably facilitates widespread bio-distribution of the trigger following development. This is evident in studies which utilize siRNAs without need for transfection reagents [57,116,117]. There are some examples of RNAi-based studies of gene suppression phenotypes in embryos including regulation of embryonic nerve cord development by *semaphorin A*, *frazzled*, and *commissureless2* [57,116,117]. However, studies designed to explore genes essential to embryonic development in mosquitoes are limited and typically utilize invasive injection methods. Further, when embryos are exposed to an RNAi trigger, the impact of knockdown is measured in later life stages. For instance, suppression of a Na^+^ methionine symporter (*AeNat5*) in *Ae. aegypti* embryos resulted in larval mortality but no impact on embryos was measured [118]. In the context of delivery, the chorion may limit direct delivery of RNAi triggers; that said, there is one example of non-invasive RNAi trigger delivery to eggs via soaking [118]. In this case, eggs were incubated in dsRNA until the time of hatching resulting in 56%–90% reduction in target gene expression in a dose-dependent manner [118]. Knockdown in the embryo also has been demonstrated indirectly through plasmid expression vectors [119,120]. Here plasmid injected into adults passed to the progeny and induced up to 99% suppression of the target gene following heat shock in progeny. There are also examples of the longevity of RNAi knockdown in adults providing up to 95% knockdown for at least 11 days post-injection in *An. gambiae* [107]. Therefore, suppression of embryo targets is potentially feasible following exposure of the parent (deemed parental RNAi or pRNAi in other insects) to embryo-specific RNAi triggers [121]. However this avenue has yet to be explored.

### 2.6. Larval & Pupal Development

Management of larval stage insects is a key component of any pest or vector control strategy. In larvae, RNAi is functional throughout the course of development with knockdown being inducible either by direct exposure or via inheritance from exposed parents [119,120,122,123]. Direct exposure of larvae has proven effective when administering RNAi triggers through injection, *per os*, soaking, or rehydration in aqueous solutions [12,13,75,123,124,125]. *Ae. aegypti* larvae soaked in an RNAi trigger for *β-tubulin* showed enhanced mortality [12]. It is not clear if soaking serves to introduce the RNAi trigger *per os*, through the cuticle, via the anal papillae or by another route. Many different delivery approaches have been used for *per os* RNAi trigger delivery in larvae, including naked dsRNA in buffer or water [126,127]. Although naked dsRNA can be internalized at high concentration in sterile conditions in the lab, dsRNA in field settings could be subject to rapid environmental degradation [21,22]. To prevent RNAi trigger degradation, abiotic and biotic delivery systems including the use of Effectene^®^ (Qiagen) liposomes, chitosan nanoparticles, *E. coli* expression systems, and *Pichia pastoris* expression systems have been explored [27,47,49,75,122,123,128,129]. Currently, at least five published studies have measured significant knockdown of 10 target genes using dsRNA encapsulated in liposomes [75,122,123,130,131]. In each case, neonate larvae were exposed *en masse* with knockdown measured at fourth instar. Chitosan is currently the most economical and environmentally safe nanoparticle delivery system for larvae. Chitosan has the added benefit of having anti-microbial activity when complexed in particles which could limit dsRNA degradation by microbes [132]. Numerous studies have utilized chitosan to form nanocomplexes with dsRNA and subsequently fed to larvae of both *Ae. aegypti* and *An. gambiae* [47,48,49,128,129]. Alternatively, both *E. coli* and *P. pastoris* expression systems facilitate even cheaper, scalable dsRNA delivery which protect dsRNA until consumption [13,27] (see Section 3.5 and Section 3.6).

For pupae, RNAi trigger exposure requires injection because pupae are a non-feeding stage. Nevertheless, injection of RNAi triggers into *Aedes, Armigeres,* and *Culex* species pupae produces knockdown of targets up to 90% [13,19,96]. Pupal development can be disrupted following direct injection of RNAi triggers for prophenoloxidase III [19,85]. Although pupae cannot be exposed *per os,* RNAi trigger exposure in larvae, or even adult female F0s could provide sufficient long term persistence to suppress pupal genes [119,120]. Indeed Mysore et al. (2014) suppressed pupal brain development following larval exposure to dsRNA chitosan nanoparticles targeting the *single-minded* gene (AAEL011013) [128].

### 2.7. Detoxification and Insecticide Metabolism/Resistance

Because RNAi can suppress detoxification and pesticide resistance genes, RNAi triggers could act as synergists to enhance insecticidal activity. Using RNAi to increase insect sensitivity to toxins was an early concept in agricultural pest control. First demonstrated in cotton bollworm, RNAi-silencing of cytochrome p450 resulted in larval susceptibility to toxic plant metabolites [133]. The use of synergists has long extended the use of many chemical pesticides and have potential for extending the use of LLINs for malaria control [134]. Although there is an abundance of literature surrounding genes identified to play roles in insecticide detoxification and desensitization, there is no immediate solution to counteract such mechanisms [7]. RNAi triggers could be applied to suppress genes which are upregulated in resistant species and strains of mosquito. Genes with p450 reductase activity as well as ABC transporters are related to detoxification and efflux of numerous insecticides [7]. In *Ae. aegypti* larvae, exposure to the oganophosphate temephos increases expression of a ABC type transporter P-glycoprotein eightfold [127]. RNAi silencing of the *P*-glycoprotein resulted in no mortality but increased larval sensitivity to temephos by ~25%.

Synergistic action of RNAi trigger knockdown is not limited to insecticide resistance genes such as noted in suppression of chitin synthase 1 in *Ae. aegypti* larval midguts. Here disruption of the peritrophic matrix facilitates uptake of diflubenzuron, calcofluor white, and dithiothreitol leading to mortality [49].

### 2.8. RNAi-Induced Pathogen Resistance

RNAi is highly attractive as a technology to parallel chemical insecticides, counter basic mosquito physiology and impact populations. Unlike chemical approaches, RNAi could also be harnessed to impact vector competence, or the inherent capacity of a mosquito to support the development and transmission of a pathogen. This concept originated in a body of mosquito-arbovirus studies that constituted the first evidence that RNAi is an antiviral innate immune response. In mosquito cells, expression of an antisense segment of the La Crosse Virus was noted as a form of “intracellular immunization” because these cells had far lower LACV titers than control cells [135]. The same was true for Dengue (DENV) or Yellow fever virus (YFV) infection [136,137,138]. This led eventually to the production of transgenic mosquitoes with RNAi-mediated resistance to DENV based on production of an RNAi trigger from a midgut-specific and blood meal inducible promoter [139,140]. Conversely, an RNAi-based approach that specifically targets virus-infected mosquitoes could be considered to selectively kill those mosquitoes that contract an infectious blood meal [141]. Disabling the RNAi machinery is lethal for virus-infected mosquitoes. Suppressing RNAi results in uncontrolled virus replication and dissemination and death of the mosquito host in *Ae. aegypti*-Sindbis virus interactions [142,143].

Although RNAi cannot be used to directly target malaria parasites developing in the mosquito host, leads for RNAi approaches to *Plasmodium* parasite control in the vector abound because the innate immune response to these parasites has been studied extensively. For example, RNAi suppression of a number of proteins involved in complement, signal modulation (e.g., serine proteases) and melanization pathways decreases numbers of midgut-stage parasites as reviewed by Blandin et al. (2008) [144]. Furthermore, Caspar, a negative regulator of an immune-responsive transcription factor, suppresses parasite numbers in the midgut, as does suppression of a midgut metalloprotease [145,146].

The mosquito-borne filarial worm parasites present yet another potential scenario for RNAi-based interventions for mosquito-borne disease control. Animal parasitic nematodes are often considered recalcitrant to RNAi-based gene suppression [147]. However, the mosquito-borne filarial worm, *Brugia malayi*, proved amenable to RNAi by injection of an RNAi trigger for a *B. malayi* cathepsin gene, with a suspected role in molting, into the mosquito host body cavity. Parasites exposed to this trigger at key life stages proved to be significantly less motile than those from control groups, and thereby are highly unlikely to be successfully transmitted [148].

## 3. Delivery Systems for RNAi Triggers In Vivo

### 3.1. Naked RNA and Modified Nucleic Acids

A variety of effective oral delivery systems using unmodified dsRNAs have been developed for larvae. In some cases larvae are soaked in large quantities of naked RNAi triggers resulting in suppression of the target after consumption [13,124,126,127]. However the aquatic environment of larvae in the field will inevitably lead to degradation of unprotected RNAi triggers [22]. Additionally the presence of midgut ribonucleases prevents successful RNAi trigger delivery in the desert locust *Schistocerca gregaria* and the German cockroach *B. germanica* [72,150]. Therefore, while oral delivery of dsRNA can successfully silence genes in mosquito larvae, knockdown may be reduced by degradation. This was noted in *Ae. aegypti* where vATPase C dsRNA was found partially degraded in tissues after 24 h [11]. The transcript was reduced by 60% but failed to kill the mosquito. Addition of chemical modifications to siRNA and dsRNA prevent degradation by nucleases without impeding RNAi. Modifications including incorporation of 2′-methoxyl-nucleotides and 5′ polyethylene glycol addition have sufficiently suppressed a Rieske iron–sulfur gene and an acetylcholine esterase gene in the diamondback moth *Plutella xylostella* [151,152]. These modified siRNAs were sufficient to induce mortality in *P. xylostella* when sprayed onto cabbage leaves demonstrating potential for modified siRNAs to be used in ATSB or other *per os* exposure applications targeting mosquito species. The ability for modified nucleotides to be delivered by *per os* exposure has been demonstrated in *Anopheles stephensi*. In this case a single stranded antisense morpholino reduced transcript of the target anti-mitogen-activated protein kinase by 60% following administration via a synthetic blood-meal [42]. These oligos are chemically altered preventing enzymatic degradation and so will not be naturally degraded in the environment but are still UV labile. While morpholino induced gene suppression is not considered ‘RNAi’ it is akin to the original interference hypothesis, and mimics early studies using anti-sense RNA to block viral transcripts [135].

In adults, oral exposure of naked dsRNA has also proven effective when exposed via a nuclease free medium. Feeding a 3%–5% sucrose solution via capillary tubes containing 3–16 mg/mL dsRNA targeting *jmtA* resulted in suppression and 47% reduction in egg production in *Ae. aegypti* [27]. In terms of environmental hardiness there are also good examples of potent naked dsRNA when attached to surfaces. In a study targeting the Colorado potato beetle larvae (*Leptinotarsa decemlineata*) potato plant leaves were coated with an aqueous solution of dsRNA targeting actin and left to dry [23]. dsRNA dried onto the leaf surface was found not only to be resistant to washing but also withstood greenhouse conditions and UV as larval mortality reached 100% at least four weeks post initial applications. These studies show that naked dsRNA may be effective in liquid or sprayed Attractive Toxic Sugar Bait (ATSB) applications.

### 3.2. Insect Plasmid Expression Systems

Plasmid expression systems offer major advantages over in vitro production of dsRNA. Firstly, expression of dsRNAs using bacterial systems have long been utilized to greatly reduce the cost of synthesis for nucleic acids and proteins. These plasmids contain a promoter followed by the target sequence of interest, a loop region, and the reverse complement of the sequence of interest, which when transcribed form a long hairpin dsRNA. Bacterial production of dsRNA still requires the dsRNA to be delivered to the mosquito following production, which has proven effective *per os* [13]. Alternatively, plasmids with insect specific promoters such as β-tubulin or actin 5C can facilitate production of the dsRNA within target cells of the mosquito [17,119,120,153,154,155,156]. Insect expression plasmids can also persist through multiple generations, with dsRNA production being inducible when using heat shock promoters [119,120]. The main drawback is delivery, since typically direct injection of embryos or adults is required to enable uptake into different tissues and bacterial expression systems release dsRNA into the gut. However, delivery of plasmids into insect cells by virtue of nanoparticle complexes using cell penetrating peptides has been demonstrated [157]. The next step will be to deliver stability expressed insect expression plasmids via a non-invasive route.

### 3.3. Nanoparticle Delivery Systems

Nanoparticle complexes can provide protection of RNAi triggers from degradation in the environment and the midgut. The composition of nanoparticles varies greatly from biomacromolecular material like chitosan or peptides to chemically produced liposomes and polyethylene glycol (PEG) [49,50,72,157]. Biomacromolecular nanoparticles are advantageous as they will be degraded rather than accumulating in the environment. Of these, chitosan has been most widely reported as an RNAi trigger delivery system for mosquitoes, with efficacy when delivered orally to larvae and or injected in pupae and adults tatgetting an array of tissues [46,47,48,49,128,129,158]. Addition of quantum dots has even led to death following oral delivery of dsRNAs suppressing several essential genes in *Ae. aegypti* larvae [46]. Cell Penetrating Peptides (CPPs) offer an alternative to chitosan worthy of exploration in mosquito systems. Cell penetrating peptides (CPPs) and inorganic complexes. CPPs as described by Meade et al. are “small cationic peptides of approximately 10–30 amino acids in length that…rapidly induce their own cellular internalization through various forms of endocytosis” [159]. RNAi trigger delivery using CPPs has been explored in a variety of forms in mammalian systems with great success [159,160]. In *Spodoptera frugiperda Sf9* cells, CPPs can facilitate delivery of plasmids [157]. In *Ae. aegypti* Aag2 cells, CPPs increase the potency of IAP1 dsRNA without increasing cytotoxicity [161]). These reports expose the potential for CPPs to deliver RNAi triggers into mosquitoes though this avenue has yet to be explored in vivo.

Chemically produced liposome nanoparticles have also been explored extensively as RNAi delivery systems. In the cockroach, *B. germanica*, naked dsRNA is degraded in the midgut following ingestion but protected when encapsulated in liposomes (GenJet, SignaGen), which subsequently facilitated uptake and knockdown of β-tubulin with 100% mortality [72]. The same is found in *Drosophila* species where neonate larvae soaked in Lipofectamine 2000 (Invitrogen) reduced RNAi target transcriptinon by 50% while soaking without lipofectamine failed to induce silencing [65]. In *Ae. aegypti* and *M. sexta* naked dsRNA and *E. coli* expressed dsRNA failed to induce RNAi silencing, but Effectene^®^ (Qiagen) liposomes achieved 90% knockdown of target genes [122]. Of all Effectene^®^ liposome feeding assays 77% knockdown was achieved on average, with up to 97% knockdown across 11 genes in *Ae. aegypti* [75,122,123,130,131]. There is some general debate about the necessity of liposomes however, since soaking of 1st instart *Ae. aegypti* with or without liposomes induced mortality when using β-tubulin, Chitin synthase I, or Heat shock protein 83 RNAi triggers [12]. Regardless of necessity, liposomes are limited in their field efficacy due to extreme cost and lack of large scale production systems.

An alternative to both chitosan and liposome systems is the utilization of PRINT particles. Here numerous different reagents, such as PEG, can be molded to form complexes with defined shapes, sizes, and charges in a highly scalable production system. Uptake and bio-distribution without toxicity have been demonstrated in larval and adult *An. gambiae* [50,51].

### 3.4. Viral Expression Systems

Considering the variety of entomopathogenic viruses already safely used as biocontrol agents mosquito viral systems offer a sophisticated means of delivering and expressing RNAi triggers directly in the mosquito cell cytosol [162]. The specificity of arboviruses to their respective arthropod hosts offer a main advantage over other expression and delivery systems [162]. The utilization of viruses as molecular tools to study or modify insects is no new concept with both Sindbis and Densovirus expression systems developed over 20 years ago [163]. The first mosquito virus expression system came in 1987 when Levis et al. [164] engineered Sindbis to express a bacterial chloramphenicol acetyltransferase in avian cells replacing 1689 nucleotides with no impact on viral propagation. When using Sindbis virus, shRNA or long hairpin dsRNA sequences can be inserted to the viral genome, enabling expression of the RNAi trigger directly in the mosquito cells. One study inserted a long dsRNA hairpin targeting vitellogenin and successfully suppressed the gene in the ovum when inoculated into *Ae. aegypti* adults [165].

Densoviruses have also been utilized as expression and delivery vectors of RNAs in larvae. Here the *Ae. aegypti* densovirus (AaDNV) genome was expressed in a plasmid containing a Pol III promoter-driven expression cassettes containing short hairpin RNA (shRNA) targeting *Ae. aegypti V-ATPase* subunit A [81]. Expression of the plasmid in *Ae. albopictus* C6/36 cells produced viral stocks that when exposed to larvae resulted in silencing of V-ATPase A and significant reduction in larval longevity.

### 3.5. Bacterial Expression Systems

A major alternative to nanoparticle complexes are direct expression systems. The main advantage of such a system is scalability and cost compared to in silico production methods. For instance, the RNase III deficient HT115 *E. coli* strain can be transformed with dsRNA plasmid expression vectors and grown in bulk worry of RNA degradation. In one study, a Pet17B plasmid containing an ampicillin resistance cassette, origin of replication, and cloned inverted repeats targeting 3 genes of interest, was mass produced in HT115 *E. coli* [36]. Purified dsRNA inverted repeats were then exposed to 2 day old *Ae. aegypti* larvae in water (soaking method) resulting in 81%–97% gene suppression of 3 target genes in larval midguts. Others take this one step further and expose mosquitoes directly to the organism producing the dsRNA. There are several lines of thought for taking on such an approach. For one, biologically produced dsRNA does not need to be expressed in the mosquito and as such does not require transfection or a mosquito specific promoter. Also dsRNA is rapidly degraded in aqueous environments [22]. This concept was originally explored by feeding dsRNA expressing *E. coli* to *C. elegans* [38]. The method was translated to mosquito larvae with remarkable efficiency. In one study, live *E. coli* expressing *Ae. aegypti* sexual dependency genes (embedded in agar pellets) were fed to larvae, resulting in sterility in up to 90% of adults [33]. Direct injection of the same dsRNAs to pupae resulted in 70%–95% gene suppression and similar levels of sterility, deeming this approach highly efficacious.

### 3.6. The Pichia pastoris Expression System

*Pichia pastoris* yeast have also been successfully transformed with pPicZB plasmid containing a long hairpin RNA sequence encoding an *Ae. aegypti* juvenile hormone acid methyl transferase gene [39]. *Ae. aegypti* fed on fermented yeasts resulted in >90% knockdown and death of larvae up to 144 h post exposure.

## 4. Conclusions

To combat the proliferation of pesticide resistant mosquito vectors and control associated mosquito-borne diseases, Integrated Vector Management and Integrated Vector Borne Disease Management programs will require alternatives to chemical pesticides [7,8]. Herein, we reviewed some key examples from hundreds of documented effective RNAi triggers that impact mosquito physiology and pathogen fitness, and thereby constitute an expansive arsenal targets for mosquito and mosquito-borne disease control strategies. We contend that RNAi could be adapted and implemented using the framework for existing vector control tools, including larvicides, contact and residual sprays, toxic baits, and LLINs. To translate RNAi to field applicability, RNAi triggers likely will need to be combined with biotic (e.g., a virus, yeast or bacterial expression system) or abiotic (e.g., nanoparticle) systems that mediate both protection and uptake of the RNAi trigger [13,27,46,47,48,49,50,51]. An RNAi approach to mosquito control offers a number of advantages over traditional chemical pesticides, including vastly improved species-specificity with diminished environmental toxicity compared with chemical pesticides.

## Figures and Tables

**Figure 2:**
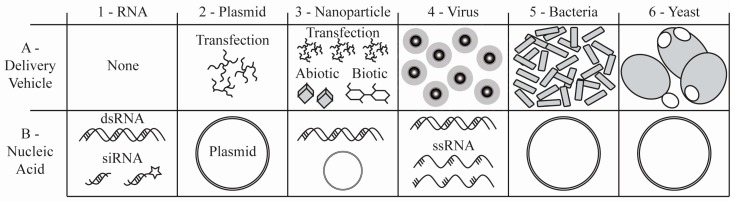
Diverse examples of mosquito RNAi trigger delivery systems (see also Table 2). The variety of (**A**) delivery vehicles and (**B**) RNAi trigger producing nucleic acids employed to suppress genes in mosquito species. Examples shown include: (**1**) naked RNAi triggers such as dsRNA, siRNA, or chemically modified siRNA (star shape); (**2**) transfection agents with dsRNA or shRNA expressing plasmids; (**3**) nanoparticles of abiotic or biotic origin in combination with dsRNA or plasmids; (**4**) viral expression systems carrying dsRNA or ssRNA that is converted to dsRNA in the cell; (**5**) bacterial expression systems containing dsRNA or shRNA plasmids; and (**6**) yeast expression systems containing dsRNA or shRNA plasmids. Numerals in the figure correspond to Section 3 of the text and Table 2.

**Table 1:** Example target genes for RNAi and phenotypic outcomes (see also Figure 1).

Legend *	Function	Gene of Interest	Accession	Outcome	Species	Reference
1	Death	Inhibitor of Apoptosis 1	AAEL009074	Death	*Ae. aegypti*	[10,52,53,54,55]
2	Olfaction & Sensation	Gustatory receptors 1 & 3	AAEL002380, AAEL010058	Inability to detect CO_2_	*Ae. aegypti*	[15]
		Odorant binding protein 1	CPIJ007604	Reduced oviposition attractant sensing	*Cx. quinquefasciatus*	[16]
3	Blood Feeding	Aegyptin	AGAP009974	Diminished blood feeding success	*Ae. aegypti*	[17]
4	Reproduction	Ovary ecdysteroidogenic hormone receptor	AAEL001915	Diminished egg development	*Ae. aegypti*	[56]
		zero population growth	AGAP006241	Spermless males	*An. gambiae*	[14]
5	Embryogenesis	Frazzled	AAEL014592	Malformed ventral nerve cord	*Ae. aegypti*	[57]
6	Larval & Pupal Development	Chitin synthase 1	AAEL002718	Disrupted peritrophic matrix	*An. gambiae*	[49]
	Morphogenesis	Prophenoloxidase III	AY487171.1	Malformed pharate adult cuticle	*Am. subalbatus*	[19]
7	Pesticide Resistance	Protease m1 zinc metalloprotease	CPIJ012471	Death by deltamethrin susceptibility	*Cx. pipiens*	[58]
8	Pathogen Uptake, Development & Transmission	Caspar	AGAP006473	Suppresses malaria parasite numbers in the midgut	*An. gambiae*	[59]

* Legend refers to both Section 2 and Figure 1.

**Table 2:** Examples of RNAi trigger delivery systems (see also Figure 2).

Legend *	(A) Delivery Vehicle	(B) Nucleic Acid	RNAi Target	Delivery Route	Reference
1	None	dsRNA	vATPase A	Adult *per os*	[11]
	None	dsRNA	P-glycoprotein	Larval *per os*	[127]
	None	siRNA	Semaphorin A	Embryo Injection	[117]
2	None	pMOS-dsRED plasmid	Aegyptin	Embryo Injection	[17]
3	Liposome (Effectene^®^)	dsRNA	Caspase 1	Larval *per os*	[123]
	Chitosan	dsRNA	Chitin synthase 1	Larval *per os*	[49]
4	Sindbis virus	Long hairpin RNA	GATA factor	Adult Injection	[149]
	Densovirus	Short hairpin RNA	vATPase A	Transfection (C6/36 cells)	[81]
5	*E. coli*	Long hairpin RNA	AAEL001684	Larval *per os* of *E. coli*	[13]
6	*P. pastoris*	Long hairpin RNA	JH acid methyl transferase	Larval *per os* of *P. pastoris*	[27]

* Legend refers to both Section 3 and Figure 2.
